# Potential Networks Regulated by MSCs in Acute-On-Chronic Liver Failure: Exosomal miRNAs and Intracellular Target Genes

**DOI:** 10.3389/fgene.2021.650536

**Published:** 2021-04-23

**Authors:** Jing Zhang, Juan Gao, Dengna Lin, Jing Xiong, Jialei Wang, Junfeng Chen, Bingliang Lin, Zhiliang Gao

**Affiliations:** ^1^Department of Infectious Diseases, The Third Affiliated Hospital, Sun Yat-sen University, Guangzhou, China; ^2^Guangdong Key Laboratory of Liver Disease Research, The Third Affiliated Hospital, Sun Yat-sen University, Guangzhou, China; ^3^Key Laboratory of Tropical Disease Control, Sun Yat-sen University, Ministry of Education, Guangzhou, China

**Keywords:** acute-on-chronic liver failure, mesenchymal stem cells, exosome, microRNA, multi-omics

## Abstract

Acute-on-chronic liver failure (ACLF) is a severe syndrome associated with high mortality. Alterations in the liver microenvironment are one of the vital causes of immune damage and liver dysfunction. Human bone marrow mesenchymal stem cells (hBMSCs) have been reported to alleviate liver injury via exosome-mediated signaling; of note, miRNAs are one of the most important cargoes in exosomes. Importantly, the miRNAs within exosomes in the hepatic microenvironment may mediate the mesenchymal stem cell (MSC)-derived regulation of liver function. This study investigated the hepatocyte exosomal miRNAs which are regulated by MSCs and the target genes which have potential in the treatment of liver failure. Briefly, ACLF was induced in mice using carbon tetrachloride and primary hepatocytes were isolated and co-cultured (or not) with MSCs under serum-free conditions. Exosomes were then collected, and the expression of exosomal miRNAs was assessed using next-generation sequencing; a comparison was performed between liver cells from healthy *versus* ACLF animals. Additionally, to identify the intracellular targets of exosomal miRNAs in humans, we focused on previously published data, i.e., microarray data and mass spectrometry data in liver samples from ACLF patients. The biological functions and signaling pathways associated with differentially expressed genes were predicted using gene ontology and Kyoto Encyclopedia of Genes and Genomics enrichment analyses; hub genes were also screened based on pathway analysis and the prediction of protein-protein interaction networks. Finally, we constructed the hub gene-miRNA network and performed correlation analysis and qPCR validation. Importantly, our data revealed that MSCs could regulate the miRNA content within exosomes in the hepatic microenvironment. MiR-20a-5p was down-regulated in ACLF hepatocytes and their exosomes, while the levels of chemokine C-X-C Motif Chemokine Ligand 8 (CXCL8; interleukin 8) were increased in hepatocytes. Importantly, co-culture with hBMSCs resulted in up-regulated expression of miR-20a-5p in exosomes and hepatocytes, and down-regulated expression of CXCL8 in hepatocytes. Altogether, our data suggest that the exosomal miR-20a-5p/intracellular CXCL8 axis may play an important role in the reduction of liver inflammation in ACLF in the context of MSC-based therapies and highlights CXCL8 as a potential target for alleviating liver injury.

## Introduction

Acute-on-chronic liver failure (ACLF) refers to the acute deterioration of liver function in patients with chronic liver disease; it is associated with high mortality rates (from 30% to 50%) among non-transplanted patients worldwide (Arroyo et al., 2020). The most common causes of ACLF in the Asia-Pacific region are chronic hepatitis B virus (HBV) infection and HBV reactivation (Sarin and Choudhury, 2016). The pathophysiological characteristics of ACLF are systemic inflammatory response and oxidative stress. Briefly, due to factors such as immune disorders and mitochondrial dysfunction, massive hepatocyte necroptosis/apoptosis occurs in a short time; therefore, persistent hepatocyte injury outpaces liver repair mechanisms and leads to inadequate liver regeneration.

Our previous finding (ClinicalTrials.gov. NCT01322906)(Lin et al., 2017) and the work of other researchers (Shi et al., 2012; Li et al., 2016) suggest that infusions with mesenchymal stem cells (MSCs) improve the liver function and decrease the mortality of patients with HBV-ACLF. This can probably be attributed to a number of factors, such as immunomodulation, acceleration of cell proliferation and angiogenesis, reduction of hepatocyte apoptosis, and anti-oxidative and anti-fibrotic effects (Francois et al., 2013; Amiri et al., 2016; Huang et al., 2016; Lee et al., 2016; Ramanathan et al., 2017). Additionally, it has also been demonstrated that human bone marrow mesenchymal stem cells (hBMSCs) improved the prognosis of HBV-ACLF humanized mice and promoted the proliferation of functional human hepatocyte lines (Yuan et al., 2019); however, the underlying mechanism remains unclear.

In the liver microenvironment, exosomes play an important role in maintaining the growth of hepatocytes; for instance, primary murine hepatocyte-derived exosomes were shown to promote the proliferation of hepatocytes in a dose-dependent manner (Nojima et al., 2015). MSCs regulate the liver microenvironment by secreting exosomes directly or other cell-mediated mechanisms indirectly, and may contribute to hepatic growth after injury (Yan et al., 2017; Liu et al., 2018). Exosomes are 30–200 nm discoid vesicles secreted by cells, which mediate cell-to-cell interactions. One of the most important cargoes in exosomes is miRNA (Redis et al., 2012; Pegtel and Gould, 2019). However, MSC-mediated changes of exosomal miRNAs in ACLF liver microenvironment are unknown.

Previous histological high-throughput studies have shown that the differentially expressed genes (DEGs) in the liver of HBV-ACLF patients (*versus* healthy liver tissues) are enriched in immune response-related genes (up-regulated)(Lin et al., 2018). The alteration of ACLF exosomal miRNAs may be a way to regulate DEGs. Therefore, here, we combined hepatic exosomal miRNA data with existing high-throughput miRNA/mRNA/protein data for the liver tissues of ACLF patients to obtain some mechanistic insights. Hub genes and their miRNAs were screened using bioinformatic tools. Overall, this work aimed to explore key exosomal miRNAs/target genes by which MSCs aid the treatment of ACLF.

## Materials and Methods

### Induction and Characterization of ACLF in Mice

The use of experimental animals in this study has been approved by the Laboratory Animal Ethics Committee of Guangzhou Forevergen Biosciences (NO.IACUC-G16020). 72 male Balb/c mice (6–7 weeks old, 20–25 g) were randomly divided into NC group and ACLF group at a ratio of 1:2, and were fed at specific pathogen-free level. For inducing ACLF, mice were intraperitoneally injected with 10% carbon tetrachloride (5 ml/kg, Sigma-Aldrich, St. Louis, MO, United States) twice a week for 8 consecutive weeks, to induce chronic liver fibrosis. Three days later, mice were intraperitoneally injected with 50% carbon tetrachloride (4 ml/kg), resulting in acute liver failure. The control group was injected intraperitoneally with the same dose of olive oil (Aladdin, Shanghai, China).

Histological assessment was performed in the livers of 6 mice in each group to characterize the ACLF model. Briefly, mice were anesthetized intraperitoneally with 4% chloral hydrate (10 ml/kg, Sangon, Shanghai, China) and euthanized 6 h after the last injection with olive oil or carbon tetrachloride. The liver tissues were then dissected and isolated, embedded in paraffin, sectioned and stained; the Sirius red, Masson’s trichrome (Sigma-Aldrich, St. Louis, MO, United States), and hematoxylin and eosin (H&E) staining techniques were used to evaluate collagen deposition, fibrosis, and inflammation and necrosis, respectively (Zhou et al., 2020).

### Isolation of Primary Hepatocytes and Co-culture System With hBMSCs

**FIGURE 1:**
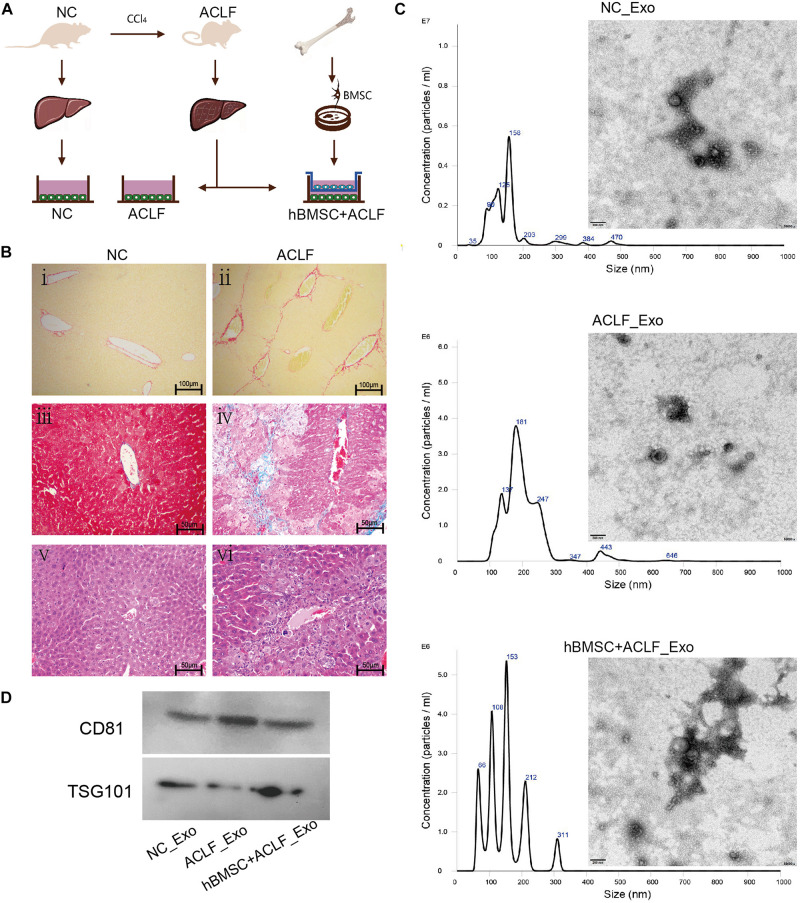
Characterization of the ACLF model and the derived exosomes. **(A)** ACLF was induced in mice using carbon tetrachloride, and then primary hepatocytes were isolated and cultured *in vitro*, in the absence or presence of MSCs. Cells from healthy animals were collected as controls. The supernatants of the three groups of cells were then collected for exosome extraction. **(B)** Sirius red staining (i-ii, scale bar = 100 μm), Masson’s trichrome staining (iii–iv, scale bar = 50 μm) and H&E staining (v–vi, scale bar = 50 μm) reveal significant fibrosis, collagen deposition, and inflammatory cell infiltration and necrosis, respectively, in the livers of ACLF mice. **(C)** NTA and TEM-based analyses of exosomes reveal that the particles are concentrated in the 200-nm range and have a cup-like bilayer structure. **(D)** Western blotting reveals the expression of the exosome membrane marker CD81 and of the intra-particle marker TSG101.

The *in vitro* model used to reproduce the hepatic microenvironment is schematized in Figure 1A. Primary hepatocytes of 24 normal mice were used as negative control (NC group). Additionally, primary hepatocytes from 48 ACLF mice were divided into two groups, the ACLF group and the MSC-treated group. Importantly, primary murine hepatocytes were isolated using a two-step perfusion method with minor modifications (Klingmuller et al., 2006). Briefly, after an intraperitoneal injection of 4% chloral hydrate, the mice were deeply anesthetized, and then their portal veins were catheterized. Then, 0.5 mM EDTA and 50 IU/ml collagenase IV (Sigma-Aldrich, St. Louis, MO, United States) preheated to 37°C were successively perfused into the liver in turns until the liver structure disintegrated; afterward, the liver capsule was opened and the liver was shaken to disperse cells. The obtained cell suspension was then filtered through a 100-μm filter (NEST, Wuxi, China) into a centrifuge tube, and the cells were washed with Willam ’E complete medium (Invitrogen, Carlsbad, CA, United States) and purified using Percoll separation solution (Biosharp, Hefei, China). Cell viability was determined by Trypan blue staining (Beyotime, Shanghai, China). Finally, primary murine hepatocytes were seeded in 6-well collagen I-coated plates (Invitrogen, Shanghai, China) at a density of 1 × 10^6^ living cells/well in HepatoZYME serum-free media (Gibco, Waltham, MA, United States). Identify primary hepatocytes through cell morphology and albumin expression.Western blotting was used to measure the expression of albumin (ab207327).β-actin (ab8227) were used as reference.

Human BMSCs were obtained from the Key Laboratory of Stem Cell and Tissue Engineering, Sun Yat-sen University. hBMSCs were characterized using previously reported protocols (Peng Y. et al., 2015). Human BMSCs at passage five were seeded at a density of 1 × 10^5^ living cells/well on a transwell filter (diameter, 0.4 μm; Corning, Corning, NY, United States) and maintained in Human MSC serum-free culture medium (Stemcell Technologies, Vancouver, Canada).

After 24 h, the culture medium in the three groups of primary murine hepatocytes was removed and fresh culture medium was added (human MSC serum-free medium (SFM) and HepatoZYME-SFM at a 1:1 ratio). In the MSC-treated group, hBMSCs pre-planted on transwell filter were co-cultured with primary hepatocytes in 6-well plates. The culture conditions were 1.5 ml SFM per well, 37°C, and 5% CO_2_. The supernatants were collected every 48 h (a total of 36 ml per group); the collection time-points were 2, 4, 6, 8, and 10 days of culture (making a total of 180 mL of cell supernatant per group). Then the cell supernatants were used to isolate exosomes.

### Isolation and Identification of Exosomes

Exosomes were isolated using the method proposed by Thery et al. (2018). Briefly, dead cells were removed by centrifugation at 2000 *g* for 20 min and cell debris were subsequently removed by centrifugation at 10,000 *g* for 30 min. An ultrafiltration membrane was then used for concentration (100 kD MWCO; Millipore, Burlington, MA, United States). Finally, the exosomes were isolated and purified using the ExO-Quick kit according to the manufacturer’s instructions (SBI Biosciences, Palo Alto, CA, United States). The resultant exosomes were then stored at −80°C until further analysis.

Exosomes were identified in accordance with the 2018 guidelines of the International Society for Extracellular Vesicles (ISEV)(Thery et al., 2018). The ultrastructure of the particles was observed under a transmission electron microscope (TEM; JEM-1400; Jeol, Tokyo, Japan). Nanoparticle tracking analysis (NTA; Nanosight; Malvern Panalytical, Malvern, United Kingdom) was used to determine the particle size and concentration. Additionally, proteins were extracted from exosomes using lysis buffer (Keygen, China) and western blotting was used to measure the levels of CD81 (exosomal membrane biomarker) and TSG101 (tumor suppressor gene 101; exosomal intramembrane biomarker); the antibodies anti-CD81 (ab109201) and anti-TSG101(ab125011) were purchased from Abcam (Cambridge, United Kingdom).

### RNA Extraction, Library Construction, and Next-Generation Sequencing Analysis of Exosomal miRNAs

In accordance with the requirements for operating the Illumina platform, the methods used in this paper were adapted from Motameny et al. (2010). The total RNA of exosomes was extracted using TRIzol (Invitrogen, Carlsbad, CA, United States). 30 ng total RNA was used for library construction. The NEBNext^®^ Multiplex Small RNA Library kit was used to construct the cDNA library (NEB, Ipswich, MA, United States). The conditions of reverse transcription are set strictly according to the manufacturer’s instructions. PCR was performed under the following cycle conditions: 94°C for 30 s, 15 cycles of 94°C for 15 s, 62°C for 30 s, and 70°C for 30 s, followed by 70°C for 5min. Reactions were run on an ABI Step One Plus Real-Time PCR system. PCR products were purified using PAGE. The constructed library was then used for quality and yield detection using an Agilent 2100 Bioanalyzer (Agilent Technologies, Santa Clara, CA, United States). Deep sequencing was performed on an Illumina HiSeq 2000 system. The raw data were analyzed using FastQC^1^. Clean reads were annotated in the miRBase (mature mouse miRNA database) to obtain the known miRNAs. MiRNA quantitation was represented as RPM (reads per million). The RPM is normalized by Z-score transformation using an R-language based online tool called OmicShare^2^. Differentially expressed exosomal miRNAs were identified according to *Z* values. The sequencing datas were deposited to the Sequence Read Archive repository^3^. Accession number is PRJNA689768.

### Multi-Omics Data From Liver Samples of ACLF Patients

Transcriptomic data were obtained from the NCBI GEO database^4^. The accession numbers used were GSE62030 (miRNA expression), and GSE14668, GSE38941, GSE62029 and GSE96851 (mRNA expression)(Farci et al., 2010; Nissim et al., 2012; Diaz et al., 2015; Chen et al., 2018). The DEGs were analyzed individually using the GEO2R web tool^5^. The miRNA screening criteria used was |logFC| ≥ 1, *p* ≤ 0.05. The mRNA screening criteria was |logFC| ≥ 2, *p* ≤ 0.05. Moreover, the DEGs from proteomics are described in the reference article (Peng L. et al., 2015).

### miRNA Target Gene Prediction

MiRNAs with consistent expression in exosomes and liver tissues were screened. For predicting the target genes of multiple miRNAs, DIANA Micro-CDS v5.0^6^ was used; the threshold was set as 0.7.

### DEG Screening

The target genes were defined as those with expression trends opposite to those of miRNAs. The Venn analysis was used to screen the target genes that were up-regulated or down-regulated in the context of transcriptome or proteome data. DEGs with statistical differences in independent studies were included in Venn analysis to ensure that the DEGs obtained in overlapping regions were statistically significant.

### Functional Annotation and Protein-Protein Interactions (PPI) Network Construction

The functional annotation of DEGs was performed using Kyoto Encyclopedia of Genes and Genomics (KEGG) analysis and gene ontology (GO) enrichment analysis. Analysis/visualization was performed using the online tool OmicShare. *p* ≤ 0.05 was taken to be significant. Additionally, PPI undirected weighted networks were constructed using STRING^7^, and clustered using Markov Clustering (MCL) with the inflation parameter set to 3. Gene expression values were conducted randomization to exclude the possibility of spurious correlations occurring by chance.

### The Network of Hub Genes and miRNAs

The molecular network topology analysis was performed using the software Cytoscape, version 3.6.1. The type of network constructed as a directed random network. The Pearson’s correlation analysis was performed for hub genes and the respective miRNAs in the microarray datasets with the accession numbers GSE62029 and GSE62030. Statistics and visualization were performed using the online tool OmicShare. The cut-off value of Pearson’s correlation analysis was set more than or equal to 0.6. *q* ≤ 0.05 was considered as significant differences.

### Verification of Gene Expression

Exosomes obtained using the abovementioned method were used to verify the expression of exosomal miRNAs. Intracellular changes in gene expression were detected in the context of a human acute liver failure cell model. Briefly, the human hepatocyte line L02 was obtained from Guangdong Key Laboratory of Liver Disease Research (the Third Affiliated Hospital of Sun Yat-sen University, Guangzhou, China) and seeded into 6-well culture plates (Corning, Corning, NY, United States) at a density of 5 × 10^5^ living cells/well, and maintained in RPMI-1640 medium containing 10% FBS and 1% penicillin/streptomycin (Gibco, Waltham, MA, United States). “Acute liver failure” was induced by treating with 0.1 mM carbon tetrachloride for 12 h (Yan et al., 2017). Then, hBMSCs were co-cultured with the injured cells for 24 h. The normal group, ALF group, and MSC-treated group were all cultured with serum-free MSC medium.

L02 cells were transfected with miR-20a-5p mimic, mimic NC, miR-20a-5p inhibitor, or inhibitor NC. After 24 h, 0.1 mM carbon tetrachloride was used to induce acute cell injury for 12 h, and the hepatocytes were co-cultured with or without hBMSC for 24 h. Collect RNA. The sequences of mimic NC (*Sense: UUCUCCGAACGUGUCACGUTT*; *Antisense: ACGUGACACGUU-CGGAGAATT*), miR-20a-5p mimic (*Sense: UAAAGUGCUUAUAGUGCAGGUAG*; *Antisense: ACC-UGCACUAUAAGCACUUUAUU*), inhibitor NC (*CAGUACUUUUGUGUAGUACAA*) and miR-20a-5p inhibitor (*CUACCUGCACUAUAAGCACUUUA*) were designed and synthesized by GenePharma Company (Suzhou, China) (Attached Table 1). Use Lipofectamine RNAiMAX (Invitrogen/Thermofischer Scientific^®^) and Opti-MEM (Gibco) to transfect siRNA, and perform transfection according to the standard procedures of the manufacturer’s instructions.

**TABLE 1:** Primer sequence for RT-qPCR.

Gene	Primer	Sequence (5′-3′)	Length
miR-20a-5p	sl	GTCCTCCTCTCCTCTCCTCTCATGAGGAGGACCTACCT	38
	F	AGGGCTAAAGTGCTTATAGTGC	22
	R	TCCTCCTCTCCTCTCCTCTC	20
U6	sl	TCGTATCCATGGCAGGGTCCGAGGTATTCGCCATGGATACGACACAAAAATATGGAACGCTT	62
	F	GTGCTCGCTTCGGCAGCACA	20
	R	TGGCAGGGTCCGAGGT	16
CXCL8 (Gene ID:3576)	F	GTGCTGTGTTGAATTACGGA	20
	R	TTGACTGTGGAGTTTTGGC	19
β-actin (Gene ID:60)	F	CATGTACGTTGCTATCCAGGC	21
	R	CTCCTTAATGTCACGCACGAT	21

*PCR, real time polymerase chain reaction; sl, stem loop; F, forward; R, reverse.*

Total RNA was extracted from the purified exosomes using TRIzol; the PrimeScript RT Reagent Kit (RR047A; Takara, Shiga, Japan), and the TB Green Premix EX Tag Kit (RR820A; Takara) were used. *U6* and β*-actin* were used as reference genes. The primer sequences are showed in Table 1. Relative expression was calculated using the comparative threshold cycle method. The data are presented as the mean ± standard error of the mean (SEM) of three independent experiments. Significant differences were analyzed using the Student’s *t-*test. The significance threshold was set at 0.05.

## Results

### ACLF Mice and Exosomes

Histopathological analysis of the liver of ACLF mice revealed pronounced collagen deposition, fibrosis, inflammatory infiltration, and necrosis (Figure 1B). The isolated primary hepatocytes are triangular in shape and express albumin. Cell purity is 95% (Supplementary Figure 1). Importantly, the characteristics of the exosomes isolated from cultured cells met the recommendation of the International Society of Extracellular Vesicles (ISEV) (Thery et al., 2018). NTA revealed particles with sizes close to 200 nm,and TEM revealed that the particles had double-membrane structures and a cup-shaped morphology (Figure 1C). Additionally, the expression of CD81 and TSG101—markers of exosomes—was clearly detected (Figure 1D).

### MSCs Impact the Abundance of Exosomal miRNAs in the Supernatants of ACLF Hepatocytes

**FIGURE 2:**
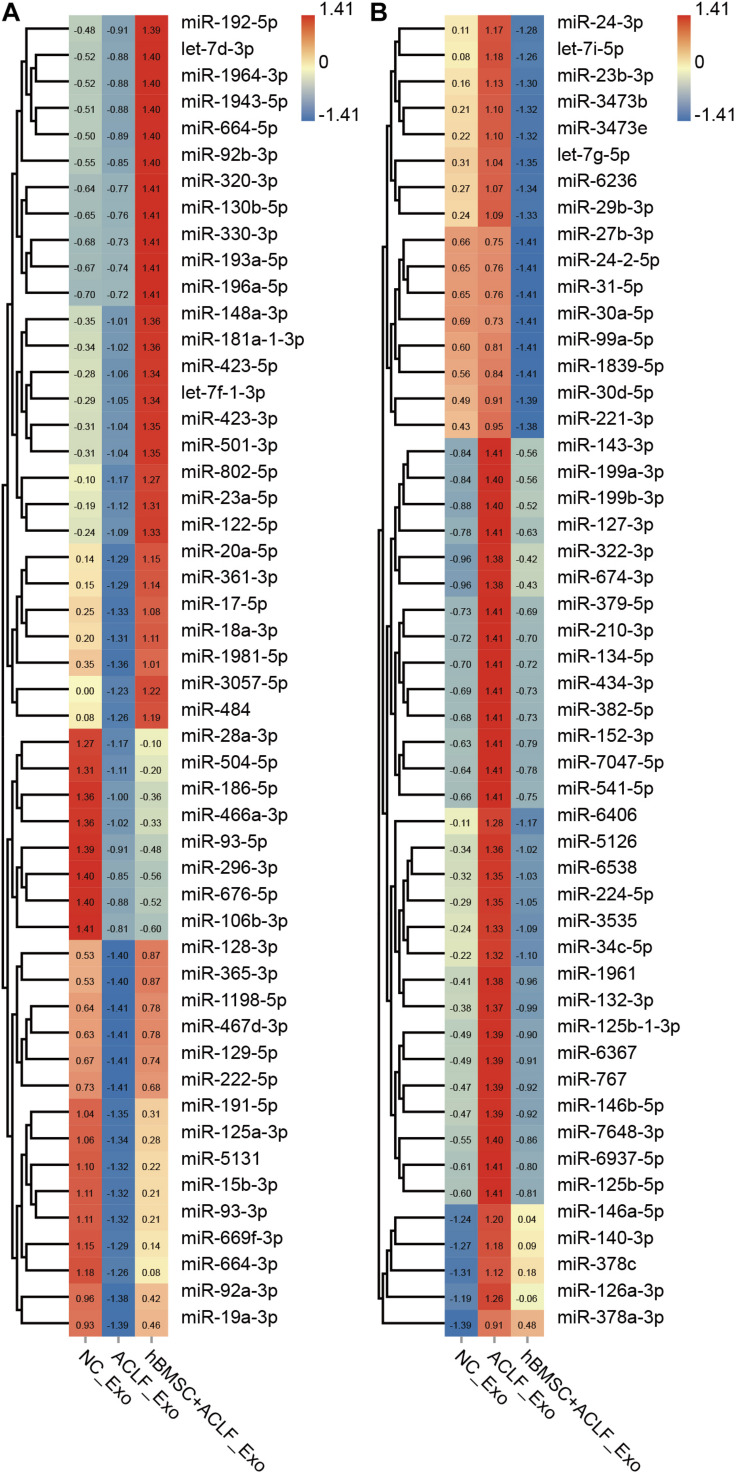
Next-generation sequencing of exosomal miRNAs. The heat map shows the relative quantification of exosomal miRNAs; 50 down-regulated **(A)** and 50 up-regulated **(B)** miRNAs in ACLF hepatic exosomes. These changes are reversed upon co-culture with hBMSCs. Clustering was performed based on the expression trend. The *Z* value was used to normalize the RPM value. Red to blue refer to high to low.

Exosomal miRNAs were quantified in the NC, ACLF, and MSC-treated groups and compared; 198 miRNAs were found to be down-regulated and 293 miRNAs were found to be up-regulated in the ACLF group (*versus* the NC group). Moreover, 90 miRNAs were found to be up-regulated and 398 miRNAs were found to be down-regulated in the MSC-treated group relative to that in the ACLF group (Supplementary Table 1). The important finding is of the miRNAs down-regulated in the ACLF group, 56 miRNAs were up-regulated in the MSC-treated group; conversely, among the miRNAs up-regulated in the ACLF group, 260 were down-regulated in the MSC-treated group. Figure 2 shows the relative quantification of 100 of these miRNAs for better display. The liver-specific miR-122-5p was down-regulated in the exosomes of the ACLF group and up-regulated in the exosomes of the MSC-treated group. It should be noted that there are two sources of exosomes in the MSC-treated group, i.e., primary murine hepatocytes and hBMSCs.

### Target DEGs of Exosomal miRNAs

**FIGURE 3:**
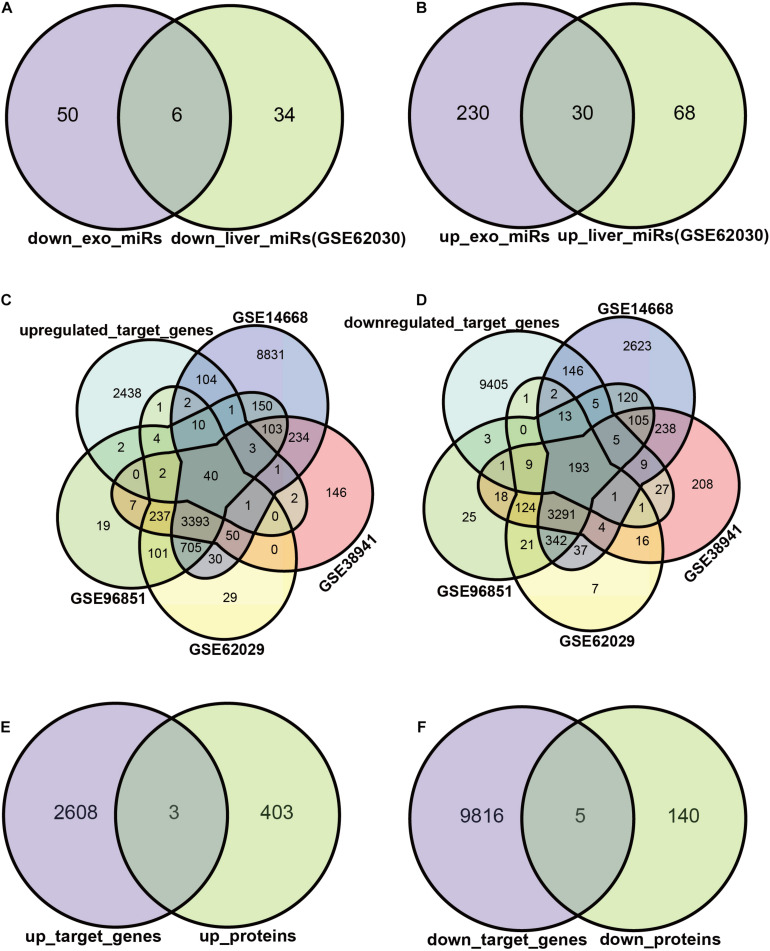
Exosomal miRNAs targeting regulated DEGs. **(A,B)** Exosomal miRNAs with identical differential expression in ACLF liver samples were screened as candidate DEmiRs. The Venn diagram shows the number of up- vs. down-regulated miRNAs. The miRNA screening criteria used was |logFC| ≥ 1, *p* ≤ 0.05. **(C,D)** The number of DEmiRs targeting genes differentially expressed in the transcriptome. The transcriptome screening criteria was |logFC| ≥ 2, *p* ≤ 0.05. **(E,F)** The number of DEmiRs target genes that are differentially expressed in the proteome. DEmiRs: differentially expressed miRNAs.

We attempted to construct exosomal miRNA-intracellular gene networks in the context of ACLF. DEGs with statistical differences in independent studies were included in Venn analysis. First, we screened the exosomal miRNAs with the same trend in ACLF liver tissues. Six miRNAs were down-regulated (Figure 3A), and 30 miRNAs were up-regulated in both hepatocytes and hepatocyte exosomes in the context of ACLF (Figure 3B). Then, target gene analysis was performed for these 36 differentially expressed miRNAs (DEmiRs): the six down-regulated miRNAs were predicted to bind to 2611 transcripts while the 30 up-regulated miRNAs were predicted to bind to 9821 transcripts. Finally, the putative target genes differentially expressed in the context of transcriptome or proteome datasets were selected as candidate target genes. The intersection of putative target genes from four ACLF liver microarray studies showed 40 up-regulated transcripts and (Figure 3C) 193 down-regulated transcripts (Figure 3D). In addition, mass spectrometry revealed the upregulation of 3 proteins (Figure 3E) and the down-regulation of 5 proteins (Figure 3F) in ACLF. Altogether, we identified 238 candidate DEGs, 233 in the transcriptome, eight in the proteome, and three in both the transcriptome and proteome.

### Biological Functions and Signaling Pathways

**FIGURE 4:**
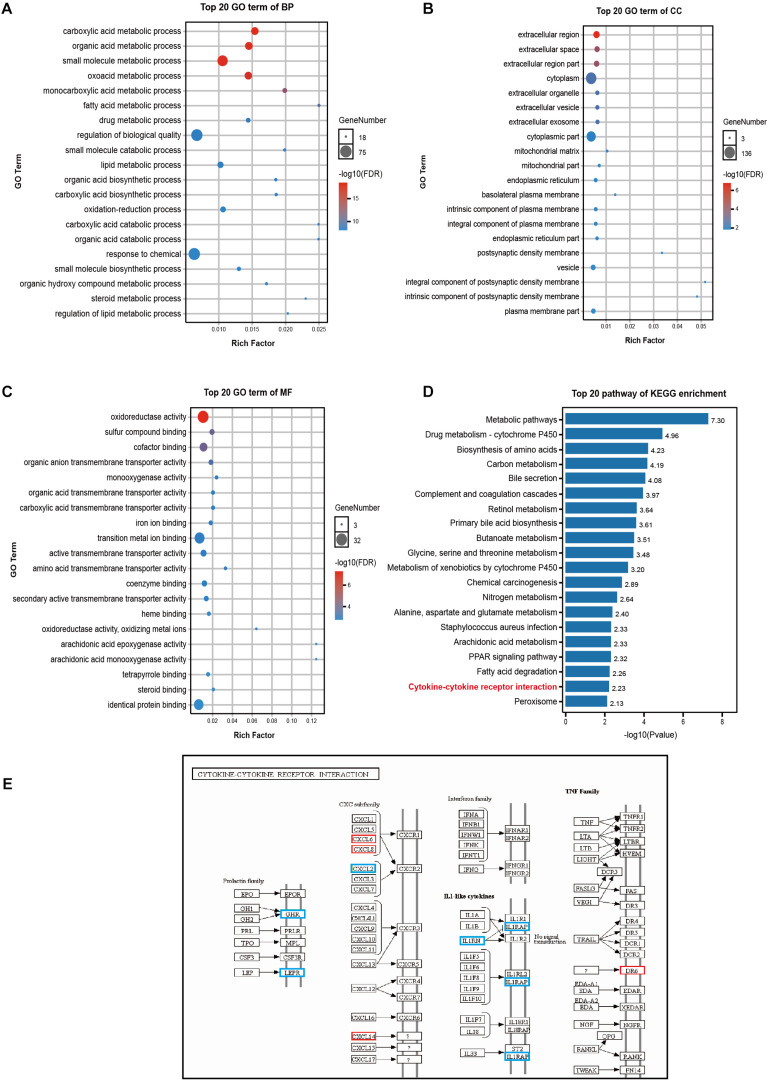
GO and KEGG pathway enrichment analyses of DEGs. **(A–C)** The 20 most significant GO terms under the BP, CC, and MF categories. The *X*-axis represents the rich ratio while the *Y*-axis highlights the GO term names. The color of the bubbles indicates the *adj. p* value. The range from red to blue represents low to high. The size of the bubbles represents the number of genes enriched in the term. BP, biological processes; CC, cellular component; MF, molecular function. **(D)** The top 20 pathways enriched as per KEGG. **(E)** DEGs of the chemokine-chemokine receptor interaction pathway. Red represents up-regulation in ACLF; blue represents down-regulation.

GO enrichment analysis revealed that DEGs affected the following biological processes (BP): small molecule synthesis, lipid metabolism, drug metabolism, and redox among others (Figure 4A). The affected cellular components (CC) involved the following: extracellular region, cytoplasm, mitochondria (Figure 4B); moreover, the following were the affected molecular functions (MF): the regulation of transmembrane protein activity and oxidoreductase activity, among others (Figure 4C). Additionally, KEGG pathway enrichment analysis revealed the involvement of the cytokine-cytokine receptor interaction pathway as well as of multiple metabolic-related pathways (Figure 4D). Nine genes were enriched for terms related to the cytokine-cytokine receptor interaction pathway (a pathway that is intimately involved in the systemic inflammatory response), of these, 5 genes were down-regulated (growth hormone receptor (*GHR*), leptin receptor (*LEPR*), C-X-C motif chemokine ligand 2 (*CXCL2*), interleukin 1 receptor antagonist (*IL1RN*), interleukin 1 receptor accessory protein (*IL1RAP*), and 4 were up-regulated *CXCL6*, *CXCL8* (also known as *IL-8*), *CXCL14*, and death receptor 6 (*DR6*, also known as the TNF receptor superfamily member 21 - *TNFRSF21*) (Figure 4E).

### Hub Genes and miRNA Networks

**FIGURE 5:**
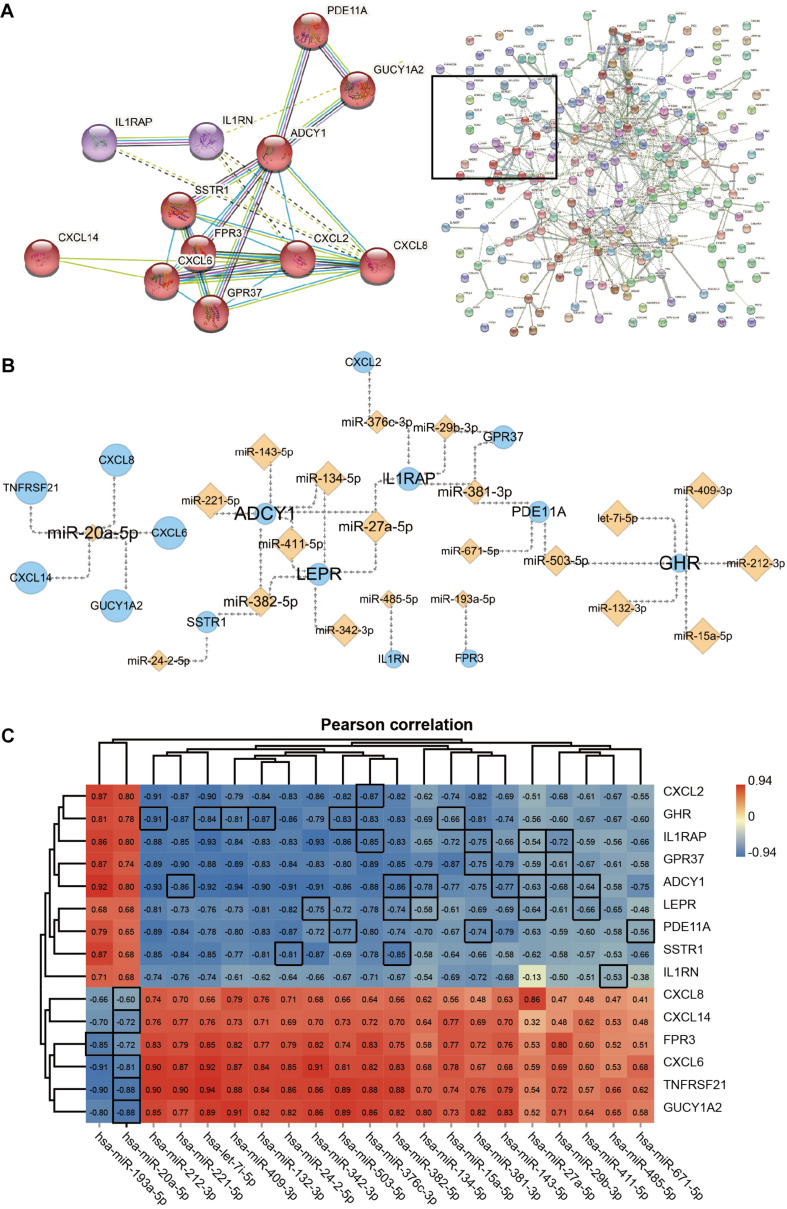
The network of Hub genes and miRNAs. **(A)** Protein-protein interaction (PPI) network and 12 DEGs in the core modules. MCL parameter set to 3. **(B)** Regulatory network of 15 Hub genes with miRNAs. Orange diamond points represent miRNAs, and blue circle points represent Hub genes. Point and label size represent the node importance index: points, from large to small, represent high to low Neighborhood Connectivity; labels, from large to small, represent high to low Degree. **(C)** Heat map showing Pearson’s correlation analysis of hub genes with miRNAs. The screened miRNAs show moderate to very strong negative correlations with their target genes. The correlation coefficients of pairs of molecules with target-regulated relationships are highlighted using black borders. Pearson correlation coefficient cut-off of ≥ 0.6. All correlation analyses in the graph are *adj. p* ≤ 0.001.

The gene cluster obtained by cluster analysis consists of chemokines and other proteins; this may be an important functional module in the PPI network which is composed of the 238 DEGs. The 12 interacting proteins were CXCL8, CXCL2, CXCL6, CXCL14, G protein-coupled receptor 37 (GPR37), formyl peptide receptor 3 (FPR3), somatostatin receptor 1 (SSTR1), adenylate cyclase 1 (ADCY1), guanylate cyclase 1 soluble subunit alpha 2 (GUCY1A2), phosphodiesterase 11A (PDE11A), IL1RN, and IL1RAP (Figure 5A). Moreover, the 15 hub genes obtained via KEGG enrichment and PPI analyses, and the corresponding 21 miRNAs together allowed the construction of the regulatory networks (Figure 5B). Topology analysis revealed that ADCY1, GHR, LEPR, and miR-20a-5p exhibited a higher Degree score; 7 miRNAs (including let-7i-5p) and 5 mRNAs (including *CXCL8*) had a higher Neighborhood Connectivity score. These molecules may serve as important nodes in the network. Last but not least, Pearson’s correlation analysis revealed a moderate to the very strong negative correlation between the expression of miRNAs and that of their target genes in ACLF (Figure 5C).

### Validation of Gene Expression

**FIGURE 6:**
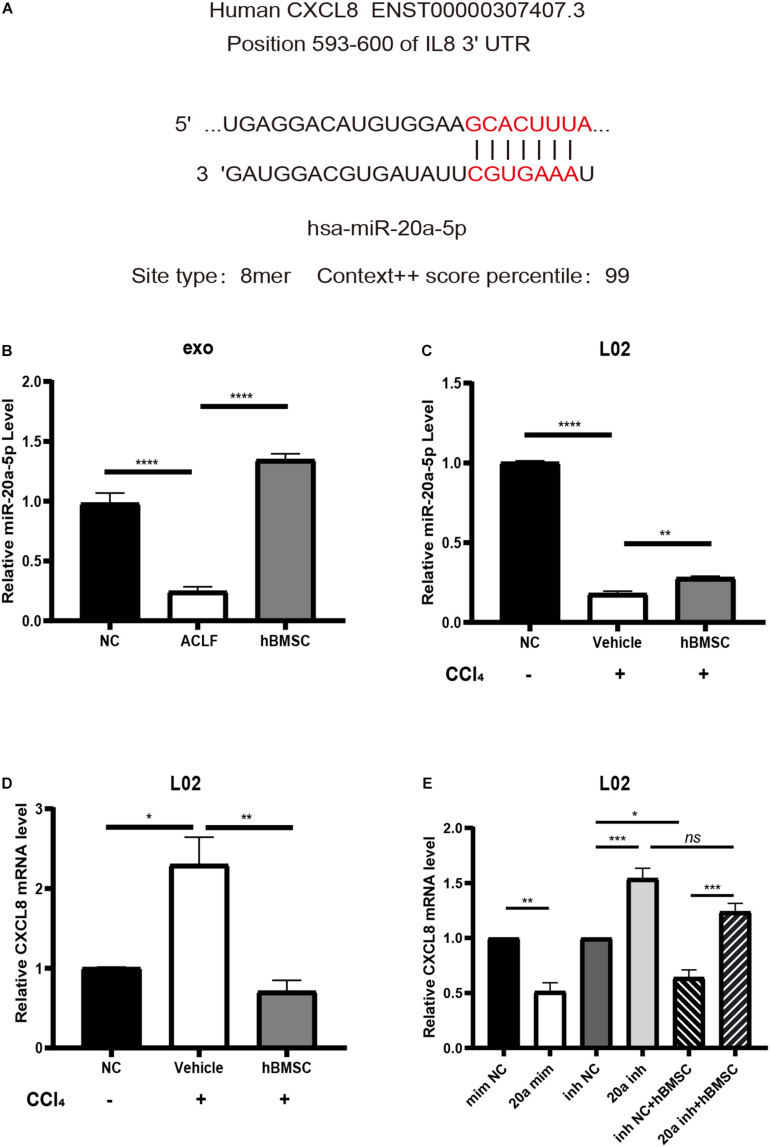
miR-20a-5p mediates hBMSCs to regulate hepatocyte CXCL8. **(A)** The binding site and targeting score of miR-20a-5p and CXCL8. **(B)** The exosomal miR-20a-5p is down-regulated in the context of hepatocytes from ACLF mice; co-culture with hBMSCs promotes its up-regulation. **(C)** Acute injury by carbon tetrachloride induces the down-regulation of miR-20a-5p in human hepatocytes (L02), while hBMSCs promote its up-regulation. **(D)** Acute injury by carbon tetrachloride induces the up-regulation of CXCL8 in L02 cells; the phenotype is reversed in the presence of hBMSCs. **(E)** Enhancement of miR-20a-5p function in carbon tetrachloride-damaged hepatocytes will lead to CXCL8 downregulation.Inhibiting the function of miR-20a-5p will lead to the up-regulation of CXCL8 and block the regulation of hbMSCs on CXCL8. CCl_4_, carbon tetrachloride. Data are represented as the mean ± SEM. All *p* values were obtained using the Student’s *t*-test: *p < 0.05, **p < 0.01, ***p < 0.001, and ****p < 0.0001. mim NC, mimic negative control; 20a mim, miR-20a-5p mimic; inh NC, inhibitor negative control; 20a inh, miR-20a-5p inhibitor.

Existing studies have demonstrated that CXCL8 can be used as a biomarker of systemic inflammatory response syndrome (SIRS); its up-regulation suggests short-term high mortality (Mikacenic et al., 2017). Therefore, we chose to verify the expression of CXCL8 and of the upstream miR-20a-5p. Targetscan analysis^8^ revealed that the 3′-UTR of the *CXCL8* transcript matched the seed sequence of miR-20a-5p in its 8*-mer* form (highest specificity), with a regulatory relationship score of 99 (Figure 6A). With respect to the levels of exosomal miR-20a-5p, the results of RT-qPCR were consistent with those of NGS. It was confirmed that exosomal miR-20a-5p in hepatocytes from ACLF mice was down-regulated, while co-culture with hBMSCs promoted its up-regulation (Figure 6B). Additionally, we used carbon tetrachloride to induce acute severe injury in L02 cells and to investigate the intracellular changes in the levels of miR-20a-5p and *CXCL8*. The results showed that the expression of miR-20a-5p was down-regulated while that of *CXCL8* was up-regulated in L02 cells under conditions of carbon tetrachloride damage. Importantly, this phenotype was reversed after 24 h of co-culture with hBMSCs (Figures 6C,D). In carbon tetrachloride-induced hepatocyte injury, CXCL8 was significantly downregulated in hepatocytes transfected with miR-20a-5p mimic. When hepatocytes were transfected with miR-20a-5p inhibitor, the CXCL8 was significantly upregulated, and hBMSC could not effectively rescue. This confirmed that hBMSC was dependent on miR-20a-5p in regulating CXCL8 in carbon tetrachloride-induced hepatocyte injury (Figure 6E).

**FIGURE 7:**
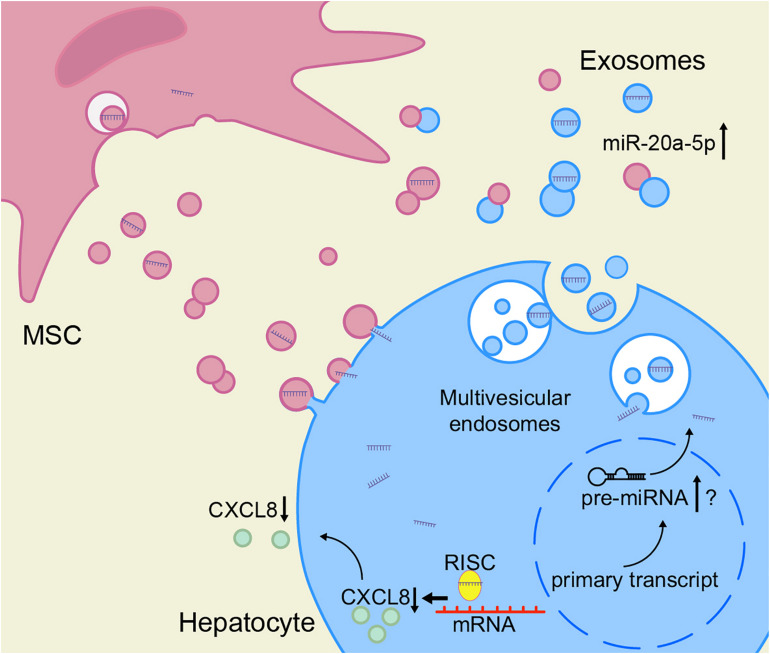
hBMSCs inhibit liver inflammation via the miR-20a-5p/CXCL8 axis. The intracellular levels of miR-20a-5p in hepatocytes increase after the uptake of miR-20a-5p-rich exosomes. In turn, miR-20a-5p inhibits the expression of CXCL8 (together with RISC), resulting in reduced CXCL8 secretion. hBMSCs not only secrete miR-20a-5p-rich exosomes directly into the hepatic microenvironment but also may promote the endogenous synthesis of miR-20a-5p in hepatocytes and its secretion as exosomes via other pathways. The accurate detection of miR-20a-5p and its precursors is required to prove the latter hypothesis. RISC, RNA-induced silencing complex.

Based on these results, we speculate that the decrease of miR-20a-5p in the hepatocytes in the context of ACLF leads to an increase in CXCL8 synthesis. To close the model, exosomal miR-20a-5p in the hepatic microenvironment increases after treatment with MSCs, leading to the inhibition of the expression of CXCL8 (Figure 7).

## Discussion

In this study, the landscape of exosomal miRNAs from primary hepatocytes of ACLF mice in the presence or absence of MSCs was determined for the first time by high-throughput sequencing. Exosomes are a major method of secretion and transport of extracellular miRNAs (Redis et al., 2012; Yu et al., 2016). Importantly, the dysregulation of exosomal miRNAs and intracellular miRNAs is thought to be associated with cell survival (Mori et al., 2019). Moreover, existing studies suggest that circulating miR-122 is a marker of hepatocyte injury, while miR-155 is a potential marker of inflammation (Li et al., 2019). In fact, the levels of plasma exosomal miR-122 are elevated in alcoholic, drug-induced, and inflammatory liver injury; in contrast, the levels of miR-122 were down-regulated in hepatocytes (Bala et al., 2012). In our findings, the levels of exosomal miR-122 were reduced in the hepatocytes of carbon tetrachloride-induced ACLF mice. This indicates that there may be a positive correlation between the distribution of miR-122 in hepatic exosomes and cells. However, another molecule closely associated with liver injury, miR-155, was found to be up-regulated in plasma exosomes and hepatocytes in the context of inflammatory liver injury (Bala et al., 2012), as well as in ACLF liver tissue microarrays. Here, in opposition to these results, we found miR-155 to be down-regulated in the liver exosomes of ACLF mice (Supplementary Table 1). Therefore, due to the selective enrichment of miRNAs, exosomal miRNAs may not always positively correlate with intracellular miRNAs.

Existing high-throughput studies in the context of ACLF lack multi-omics combined analyses. In this study, 36 dysregulated miRNAs and 238 DEGs were successfully identified via the analysis of dysregulated exosomal miRNAs and hepatocytes miRNA/mRNA/proteins in the context of ACLF. miRNA-mediated gene expression abnormalities have been identified in immune destabilization, oxidative stress, programmed death, and other processes (Guicciardi et al., 2013; Hirschberger et al., 2018; Klieser et al., 2019). Given the recovery of dysregulated exosomal miRNAs after treatment with MSCs, we attempted to identify key molecular targets among the target genes of miRNAs. As per PPI network analysis, cytokine-cytokine receptor interaction may be an important functional module among 238 DEGs. It is worth noting that KEGG enrichment analysis produced a similar result; 238 DEG were significantly enriched in the context of cytokine - cytokine receptor interactions. Previous reports suggest that ACLF is a cytokine and chemokine-induced systemic inflammatory state (Arroyo et al., 2020). In short, MSCs may regulate the levels of synthesis and secretion of chemokines by hepatocytes through exosomal miRNAs.

The uncontrolled activation of intrinsic immunity is a prerequisite for the development of systemic inflammation in ACLF. The chemokine cascade is a key step in the recruitment and activation of neutrophils in the liver of ACLF patients (Casulleras et al., 2020). The pro-inflammatory chemokine CXCL8 is produced by liver cells including hepatocytes, stellate cells, and Kupffer cells (Arango Duque and Descoteaux, 2014). In addition, the levels of CXCL8 are higher in the liver of ACLF patients than in their peripheral blood (Khanam et al., 2013); this is probably responsible for the migration of neutrophils into the liver in a CXCR1/2-dependent manner, and their consequent activation (French et al., 2017). Notably, studies have reported that CXCL8 levels are elevated in the serum of patients with ACLF or acute liver failure; in fact, the levels of CXCL8 significantly correlate with the serum levels of total bilirubin, and are significantly associated with high MELD scores and short-term mortality (Khanam et al., 2013; Bonkovsky et al., 2018; Trebicka et al., 2019). This suggests that lowering the levels of CXCL8 may serve as a strategy for the treatment of ACLF. Importantly, in our study, MSC therapy was found to increase the expression of miR-20a-5p and inhibit the synthesis of CXCL8 in hepatocytes, and the regulation of CXCL8 depends on miR-20a-5p. In fact, this is in line with previously published results showing that miR-20a up-regulation is associated with the inhibition of CXCL8 expression and attenuation of high glucose-induced inflammation and apoptosis in renal tubular cells (Bai et al., 2020). This was also true in the context of stromal fibroblasts (Signs et al., 2018). Altogether, these results suggest that the miR-20a-5p/CXCL8 axis may be an important target for the treatment of ACLF and that the use of MSCs is a potential approach.

The down-regulation of hepatic miR-20a-5p is associated with the development of inflammation and fibrosis (Fu et al., 2020), while the up-regulation of miR-20a-5p is associated with the promotion of hepatic glycogen synthesis (Fang et al., 2016), reduction of lipid hyperaccumulation (Wang et al., 2020) and promotion of hepatocyte proliferation (Chen et al., 2016). Therefore, miR-20a-5p may regulate multiple targets to promote the repair of the injured liver. We unexpectedly found in our results that miR-20a-5p also negatively regulates DR6 (TNFRSF21). Interestingly, DR6 induces apoptosis, either through the activation of NF-κB, or via Bax (Zeng et al., 2012; Hu et al., 2014). Therefore, miR-20a-5p expression—elevated in exosomes as well as in hepatocytes in response to MSC treatment—in the context of ACLF, may cause a decrease in the expression of DR6 in hepatocytes and thus reduce their apoptosis.

It would be interesting to investigate whether the up-regulated exosomal miR-20a-5p originated from hBMSCs or hepatocytes. According to published data, miR-20a-5p was detected in hBMSC-derived exosomes, accounting for 0.11% of total exosomal miRNAs (Ferguson et al., 2018). Remarkably, in our study, miR-20a-5p accounted for 0.19% of the total exosomal miRNA in the normal murine hepatocytes (Supplementary Table 1); moreover, in the GSE62030 dataset, miR-20a-5p accounted for 0.2% of the total hepatic miRNA in the healthy group (Diaz et al., 2015). In addition, in our another study, we found that miR-20a-5p was increased in the liver tissues of mice with LPS/D-gal induced acute liver failure, 3, 6, and 12 h after infusion of hBMSC-derived exosomes. The increase in miR-20a-5p was also observed in the context of L02 cells treated with hBMSC-exosomes *in vitro* (data not shown). Therefore, it is quite possible that MSCs deliver miR-20a-5p to hepatocytes through their own exosomes. However, we cannot exclude the possibility that MSC-derived exosomes promote endogenous miR-20a-5p synthesis in hepatocytes through unknown mechanisms. The accurate detection of miR-20a-5p as well as of its precursors should be performed to clarify the above doubt.

This study is not without limitations. The main ones are the lack of high-throughput data on exosomal miRNAs from ACLF patients; we need to bear in mind that there may be differences in the expression of exosomal miRNAs in human-derived *versus* murine-derived hepatocytes in the context of ACLF. The use of ACLF humanized mice or the direct use of primary hepatocytes obtained from ACLF patients after transplantation would be a better option; however, there are technical and ethical limitations for the time being. The functional analysis of exosomal miRNAs may be important for the understanding of the hepatic pathological mechanisms and/or for the identification of the therapeutic targets (Sato et al., 2016). In any case, high-throughput screening and bioinformatic analysis could only provide directions and targets for the study on molecular mechanisms *per se*; therefore, the predicted molecular targets still need to be functionally validated *in vitro* in human liver cell lines as well as *in vivo*, in animal models. Importantly, the miRNA profile in the context of exosomes from the hepatocytes of an ACLF murine model identified in this study provides important information to facilitate future studies on high-throughput multi-omics analyses regarding the pathogenesis and treatment of ACLF.

Right now, no consensus exist on the optimal method of MSC delivery (Caplan et al., 2019). There is no difference in efficacy of MSCs administered by peripheral vein, hepatic artery, portal vein, or intrahepatic injection (Alfaifi et al., 2018). Another difficulty in the clinical application of MSC is that MSC from different tissues all carry different levels of highly procoagulant tissue factor (TF), which triggers an immediate blood-mediated inflammatory response and induces thrombosis after cell transplantation. Although BM-derived MSCs have better blood compatibility, there is still a need to develop new alternatives, such as MSC-derived exosomes (Moll et al., 2019). The new “cell-free” approach is expected to solve the limiting factors of MSC clinical application, such as thrombosis and low survival rate of transplanted cells.

## Conclusion

This study is the first exploration on hepatic exosomal miRNAs in the context of ACLF in the presence or absence of MSCs. A transmembrane negative regulatory network was constructed on the basis of existing multi-omics data on ACLF. The genes involved could help us to explain the immunomodulatory effects observed upon the administration of MSCs in the context of ACLF; MSCs promote the elevation of miR-20a-5p levels in hepatocytes and exosomes leading to the consequent decrease in the expression of CXCL8 in hepatocytes. We believe these data will contribute to future molecular mechanistic studies as well as to the development of translational applications of MSCs for treating ACLF.

## Data Availability Statement

The datasets presented in this study can be found in online repositories. The names of the repository/repositories and accession number(s) can be found in the article/Supplementary Material.

## Ethics Statement

The animal study was reviewed and approved by Laboratory Animal Ethics Committee of Guangzhou Forevergen Biosciences.

## Author Contributions

All authors listed have made a substantial, direct and intellectual contribution to the work, and approved it for publication.

## Conflict of Interest

The authors declare that the research was conducted in the absence of any commercial or financial relationships that could be construed as a potential conflict of interest.

## Footnotes

1 http://www.bioinformatics.babraham.ac.uk/projects/fastqc/

2 http://www.omicshare-com/tools

3 https://www.ncbi.nlm.nih.gov/sra

4 http://www.ncbi.nlm.nih.gov/GEO/

5 http://www.ncbi.nlm.nih.gov/geo/geo2r/

6 http://diana.imis.athena-innovation.gr/DianaTools

7 https://string-db.org/

8 http://www.targetscan.org/vert_72/

## Supplementary Material

Supplementary Figure 1 Characterization of primary murine hepatocytes. **(A,B)** The primary hepatocytes of mice in the NC group and ACLF group were triangular in shape under light microscope. Scale bar = 400μm. **(C,D)** Western blot detection of ALB expression in mouse primary hepatocytes. The relative expression of ALB in primary hepatocytes of the NC group was 95% of that in the AML12 mouse hepatocyte line.

## Abbreviations

ACLF: acute-on-chronic liver failure
ADCY1: adenylate cyclase 1
BP: biological processes
CC: cellular components
CCl4: carbon tetrachloride
CXCL: C-X -C motif chemokine ligand
DEGs: differentially expressed genes
DEmiRs: differentially expressed miRNAs
DR6: death receptor 6
exo: exosomes
FPR3: formyl peptide receptor 3
GHR: growth hormone receptor
GO: gene ontology
GPR37: G protein-coupled receptor 37
GUCY1A2: guanylate cyclase 1 soluble subunit alpha 2
hBMSCs: human bone marrow mesenchymal stem cells
IL1RAP: interleukin 1 receptor accessory protein
IL1RN: interleukin 1 receptor antagonist
KEGG: Kyoto Encyclopedia of Genes and Genomics
LEPR: leptin receptor
MF: molecular functions
NGS: next-generation sequencing
NTA: nanoparticle tracking analysis
PDE11A: phosphodiesterase 11A
PPI: protein-protein interaction
RISC: RNA-induced silencing complex
RPM: reads per million
SSTR1: somatostatin receptor 1
TEM: transmission electron microscope
TNFRSF21: TNF receptor superfamily member 21.
