# Supplementary figures and images for: Potential Networks Regulated by MSCs in Acute-On-Chronic Liver Failure: Exosomal miRNAs and Intracellular Target Genes
Source: Front Genet. 2021 Apr 23;12:650536. doi: 10.3389/fgene.2021.650536 (PMC8102832; doi:10.3389/fgene.2021.650536)

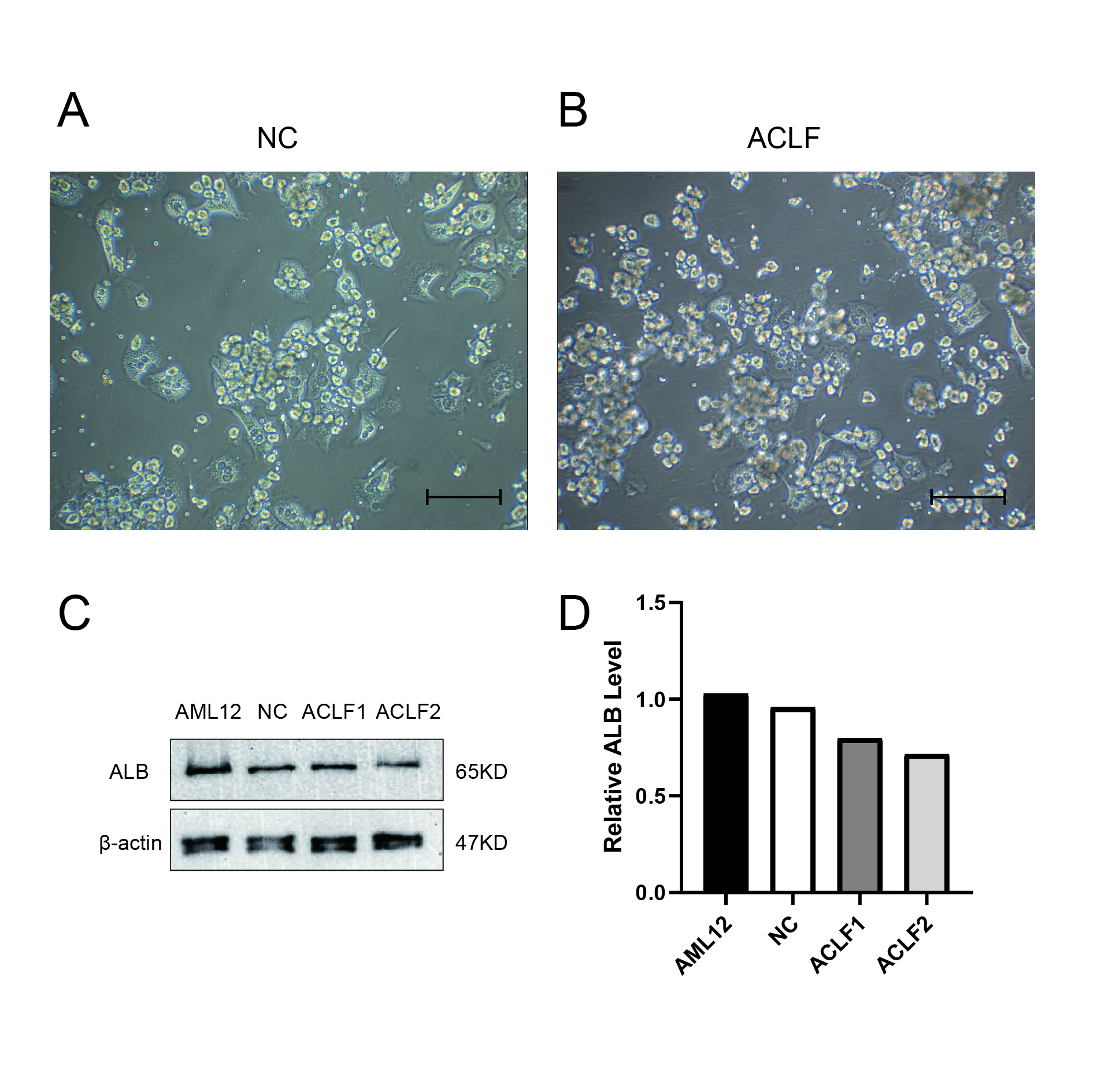

Supplement: Supplementary Figure 1 — Characterization of primary murine hepatocytes. (A,B) The primary hepatocytes of mice in the NC group and ACLF group were triangular in shape under light microscope. Scale bar = 400μm. (C,D) Western blot detection of ALB expression in mouse primary hepatocytes. The relative expression of ALB in primary hepatocytes of the NC group was 95% of that in the AML12 mouse hepatocyte line. [file Image_1.JPEG]
